# Influence of the Second-Phase Resin Structure on the Interfacial Shear Strength of Carbon Fiber/Epoxy Resin

**DOI:** 10.3390/ma17061323

**Published:** 2024-03-13

**Authors:** Hansong Liu, Jinsong Sun, Lianwang Zhang, Zhaobo Liu, Chengyu Huang, Mingchen Sun, Ziqi Duan, Wenge Wang, Xiangyu Zhong, Jianwen Bao

**Affiliations:** 1 AVIC Manufacturing Technology Institute Composite Technology Center, Beijing 101300, China; liuhansongzhfc@foxmail.com (H.L.); sunjsbuaa@163.com (J.S.); zhanglian51@163.com (L.Z.); 2 School of Materials Science and Engineering, Beihang University, Beijing 100191, China; lzbbuaa@163.com (Z.L.); huangchengyu@buaa.edu.cn (C.H.); smc703@126.com (M.S.); 3 AVIC Composite Corporation Ltd., Beijing 101300, China; dzq970920@163.com (Z.D.); 15966308692@163.com (W.W.)

**Keywords:** carbon fiber, epoxy resin, surface microstructure, interfacial shear strength, hygrothermal treatment

## Abstract

The toughening modification of epoxy resin has received widespread attention. The addition of the second-phase resin has a good toughening effect on epoxy resin. In order to investigate the effect of the second-phase resin on the interphase of composites, in this work the interfacial properties of carbon fiber (CF)/epoxy resin with the second-phase resin structure were investigated. Methodologies including surface structure observation, chemical characteristics, surface energy of the CF, and micro-phase structure characterization of resin were tested, followed by the micro-interfacial performance of CF/epoxy composites before and after hygrothermal treatment. The results revealed that the sizing process has the positive effect of increasing the interfacial bonding properties of CF/epoxy. From the interfacial shear strength (IFSS) test, the introduction of the second phase in the resin reduced the interfacial bonding performance between the CF and epoxy. After the hygrothermal treatment, water molecules diffused along the interfacial paths between the two resins, which in turn created defects and consequently brought about a reduction in the IFSS.

## 1. Introduction

As a kind of traditional thermosetting material, epoxy resins can form a crosslinked mesh structure under the action of a curing agent. The resulting resins have good dimensional stability, heat resistance, abrasion resistance, electric insulation, etc. [1,2,3,4,5], and are widely used in industries such as coatings, aerospace, electrical and electronics, insulating materials, adhesives, and more [6,7,8,9]. However, typical problems of conventional epoxy resins after curing include brittleness, poor impact resistance, vulnerability to cracking, etc., which can make it difficult to meet the application requirements of aerospace and other engineering and technical industries.

In order to improve the ability of composites to resist damage formation and growth, the toughening modification of epoxy resin has received extensive attention from researchers [10,11,12,13,14,15]. There are many ways to toughen epoxy resin, among which the more common include rubber-toughened epoxy resin [16], thermoplastic resin-toughened epoxy resin [17], curing agent containing flexible chain-toughened epoxy resin [18], core–shell structure polymer-toughened epoxy resin [19], thermotropic liquid crystal material-toughened epoxy resin [20], IPN structure-toughened epoxy resin [21], nano-particle-toughened epoxy resin [22], and hyperbranched polymer-toughened epoxy resin [23]. Among these methods, researchers have found that the addition of the second-phase resin has a good toughening effect on epoxy resins.

In terms of the toughening mechanism, researchers have successively proposed various epoxy resin toughening theories, such as the tearing and plastic stretching mechanism of the dispersed phase, the cracking mechanism of the passivated matrix resin, the cracking and riveting mechanism, and the over-exfiltration theory [24,25], among others. The toughening mechanism can be divided in terms of phase behavior into a phase separation mechanism [26] and non-phase separation mechanism [27,28]. Phase separation occurs during toughening with the second-phase resin. Inoue et al. [29] proposed reaction-induced phase separation in their study of rubber-toughened epoxy resins and pointed out that the best toughening effect could be obtained when rubber particles formed a mono-dispersed phase structure in the blending system, which can be well explained by the tearing and plastic tensile mechanism of the dispersed phase. Therefore, the current research focuses on the phase separation toughening system. This type of second-phase resin surface either does not contain functional groups or contains only a single functional group (such as hydroxyl, carboxyl, etc.). The matrix epoxy resin is poorly compatible with the second-phase resin, resulting in a chemically-induced phase separation, with the second phase of the resin in the form of particles becoming dispersed in the epoxy matrix to form a kind of discontinuous structure.

Although there have been many research papers on resin toughening modification [30,31,32], there is still a lack of research on the interface between epoxy resin and carbon fibers (CFs) for CF-reinforced resin matrix composite systems, which are currently used in a large number of applications. For such a CF and resin matrix, a large number of researchers have focused on CF, carrying out a huge number of modifications on the surface of CF and composites [33,34,35,36,37,38,39]. However, many of these works can only be used for small specimens, and cannot be used for continuous production. On the other hand, the interfacial behavior of modified epoxy resins toughened with CFs, which are heavily used in engineering, has not received as much attention from researchers. Ilyin et al. [40,41] researched the impact toughness of epoxy–nanofiller matrices and the adhesion between aramid fiber or CF and an epoxy matrix containing different nanoparticles, demonstrating the relationship between the strength of the fiber/matrix adhesion and the strength of the resulting composite reinforced with aramid fiber or CF. It is apparent that the interfacial issue is very important for composite manufacturers during material selection, program development, etc.

In this work, we have investigated the effect of the second-phase resin structure on the interfacial bond strength of a CF/epoxy resin. First, the basic properties of CFs were characterized in detail, focusing especially on the surface microstructure of the CFs, and a computer-assisted calculation method was used to quantitatively characterize the groove structure of the CF surface. On this basis, the second-phase component in the epoxy resin was observed in order to comprehensively evaluate the interaction effect between CF and epoxy resin. Finally, the interfacial bonding of the CF/epoxy resin was evaluated, with the effect of introducing the second phase on the mechanical properties of the CF/epoxy resin interface reflected by calculation of the interfacial shear strength (IFSS) and interfacial fracture toughness. In addition, the law of fiber–resin bonding characteristics was investigated through the hygrothermal environment test. This study provides a theoretical reference for the application of modified epoxy resins in CF-reinforced resin matrix composites.

## 2. Materials and Methods

### 2.1. Materials

The resin matrix used in the composite was the AC53X epoxy series (AVIC Manufacturing Technology Institute Composite Technology Center, Beijing, China). The second-phase resin named RS2000 (molecular weight ~30,000) was supplied by the AVIC Manufacturing Technology Institute Composite Technology Center (Beijing, China). The reinforcement (CF800, T800 grade) was supplied by Weihai Tuozhan Fiber Co., Ltd. (Weihai, China). The solvent acetone was purchased from Beijing Chemical Works (Beijing, China). Deionized water was bought from Beijing Fengyuhua Co., Ltd. (Beijing, China). Ethylene glycol and formamide were purchased from Xilong Scientific Co., Ltd. (Shantou, China). Specific information on the materials can be found in the Supplementary Materials.

### 2.2. Preparation

In order to investigate the effect of the CF surface state on the bond strength of the CF/epoxy interface, the sizing agent on the CF surface was removed by heat treatment. Based on the results of a previous study [42], the heat treatment condition was set at 350 °C for 30 min in air. The oven for heat treatment was numbered as HD-100B (Shanghai Shibei Instrument and Equipment Factory, Shanghai, China). The sizing content (*SC*) of each sample was calculated according to Equation (1):

(1) $$SC=\frac{m_{0}-m_{1}}{m_{0}}\times 100\%\;$$

where $m_{0}$ is the initial mass of the sample and $m_{1}$ is the mass after heat treatment of the sample.

For AC53X-1, the second-phase resin was added at a level of 12.5%. The mixing conditions were 80 °C, 20 min, and 100 rpm. For AC53X-2, no second-phase resin was added.

### 2.3. Tests and Measurements

The morphology of the samples was evaluated via field emission scanning electron microscope (SEM, Quanta 450 FEG, FEI, Hillsboro, OR, USA) after metal sputtering and by atomic force microscope (AFM, Veeco D3000, Bruker Corporation, Billerica, MA, USA). Based on the SEM images of the CF, the method of statistical calculation of the grooves was applied. Five images of each sample were processed using MATLAB (Version R2020a), and the contour shape of the CF cross-section was extracted. The number, depth, and width of the grooves on the surface of the CF were calculated using the program.

The surface element content of the samples was investigated with an energy dispersive spectrometer (EDS, OXFORD X-Max, Oxford Instruments, Oxford, UK) and X-ray photoelectron spectroscopy (XPS, Thermo VG ESCALAB 250, Thermo Fisher Scientific, Waltham, MA, USA) tests. The contact angle between the CF and specific liquid was observed using dynamic contact angle measure equipment (DCAT25, Dataphysics, Stuttgart, Germany) with a test speed of 0.02 mm·s^−1^. The microphase structure of the resin was observed using a metallographic microscope (LEICA DMLM, Leica, Wetzlar, Germany). The IFSS test was conducted to explore the interfacial bonding properties of the CF/epoxy. Curing of the resin was carried out in an oven at 180 °C for 2.5 h. The resulting specimen was a CF monofilament with a diameter of 5 μm and diameter of the resin microsphere of 40 μm. The IFSS was measured by pulling off the cured resin droplet from the CF monofilament. The IFSS of each sample was calculated according to Equation (2):

(2) $$IFSS=\frac{F}{\pi dl}\;$$

where *F* is the maximum force, *d* is the average diameter of the CF, and *l* is the length of the CF embedded in the resin droplet.

The interfacial fracture toughness (*G_ic_*) of the CF/epoxy was calculated using Equation (3). The dimension of the specimen was the same as for the IFSS test. Based on analysis of the variational mechanics and shear–lag, and assuming that the resin is spherical, the *G_ic_* can be calculated as follows [43]:

(3) $$G_{ic}=\frac{4\tau^{2}r_{2}^{2}}{r_{1}E_{1}}+\frac{12\tau^{2}r_{1}r_{2}^{2}}{E_{2}(2r_{2}^{2}-3r_{1}^{2})}$$

where *E*_1_, *E*_2_, *r*_1_, and *r*_2_ are the Young’s modulus of the fiber (~295 GPa), the Young’s modulus of the epoxy resin droplet (~4 GPa), the radius of the fiber (~5 μm), and the radius of the epoxy resin droplet (~40 μm), respectively. To test the resistance of the specimens to moisture and heat, the specimens were placed in a thermostatic water bath (HH-W600, OLABO, Jinan, China) at a treatment temperature of 71 °C and immersed in water for 1 day, 3 days, and 5 days. The dimensions of the specimens were the same as for the IFSS test.

## 3. Results and Discussion

### 3.1. Surface Microstructure of CFs

The surface microstructures of the CFs were characterized to provide a reference for the subsequent numerical analysis of the interfacial bond strength. The pristine CFs and desized CFs were labeled as CF800 and CF800Q, respectively. The *SC* of the CFs after treatment according to the desizing conditions provided in Section 2.2 was 0.9%, which is close to the value provided by the CF manufacturer.

**Figure 1:**
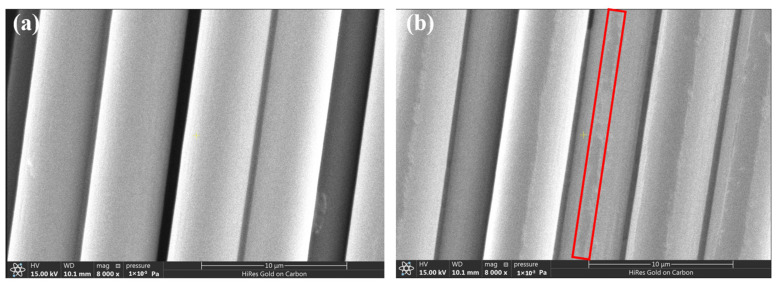
Surface microstructures of (**a**) CF800 and (**b**) CF800Q.

Figure 1 exhibits the surface morphologies of CF800 and CF800Q. The SEM images reveal that the CF has a smooth interface before desizing, which indicates weak physical interfacial adhesion between the matrix and CFs due to a lower number of mechanical engagement sites. On the other hand, the surfaces of the desized CFs show traces produced by the decomposition of the sizing agent while undergoing heat treatment, which are distributed along the axial direction of the fibers. From Figure 1, it can be seen that the desizing initially affects the surface morphology of the CFs. In addition, the sizing agent is more chemically active than the CF, as it is a thin layer of uncrosslinked oligomers. After removing this active layer, the chemical composition of the CF surface is bound to change. The changes in these parameters all have an effect on the interfacial bonding of the CF/epoxy. Therefore, these factors were systematically characterized and analyzed by subsequent tests.

**Figure 2:**
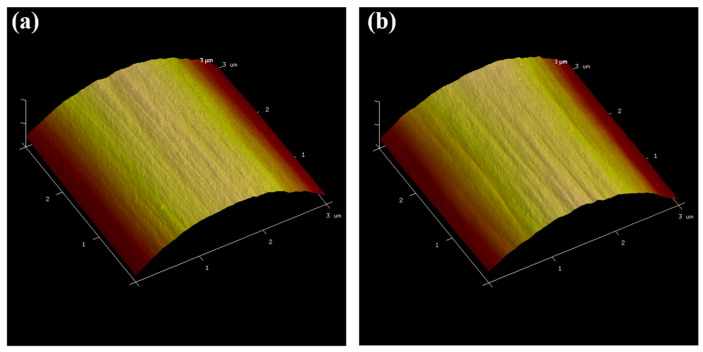
AFM images of (**a**) CF800 and (**b**) CF800Q.

The detailed surface structures and roughness values of CF800 and CF800Q were characterized by AFM. It can be seen from the AFM images in Figure 2 that the CFs present a shallow groove structure on their surfaces. While the surface of the CF in the SEM image is smooth, on a finer scale it can be seen that the surface of the CF has a structure of shallow grooves along the axial direction. Comparing the AFM images of CF800 and CF800Q, it can be seen that the depth of the grooves on the surface of CF800Q is deeper. This appearance indicates that the sizing agent on the surface of the CF covers the original groove structure of the CF to a certain extent, which makes the surface of the CF smooth. To verify this judgment, the surface roughness (root mean square roughness R_q_, average of five samples) of the CFs was calculated using Nano Scope Analysis software (Version 1.0.0.0.0), with the results shown in Table 1. It can be seen that, corresponding to the AFM images, the roughness value of CF800Q has no obvious difference from that of CF800.

**Figure 3:**
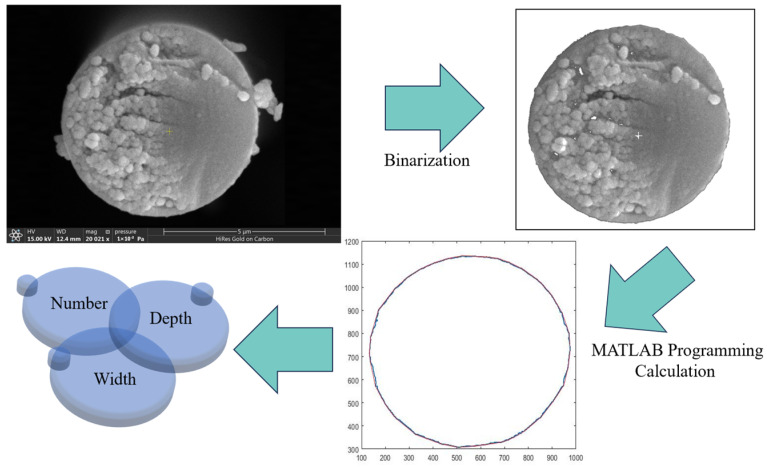
Schematic diagram of quantitative characterization of CF cross-section.

Although AFM can reflect the detailed structural information of the CF surface, its test range is only a very small localized area on the CF surface, meaning that the characteristics of its response are not very representative and may not reflect the physical structure law of the CF surface. In addition, the roughness obtained by AFM is the average value of roughness in a specific small area, which cannot well reflect the characteristics of groove shape and depth. Based on the above problems, a more in-depth analysis of the surface microstructures of the CF was carried out by statistical calculation of the grooves, as shown in Figure 3. The CF cross-section image was processed using MATLAB; the contour shape of the CF cross-section was extracted after removing the surface impurities, and the number, depth, and width of the grooves on the CF surface were calculated by the program to realize the quantitative characterization of the grooves on the surface. The results are shown in Table 2.

As can be seen in Table 2, the surface groove structure of CF800 changes after desizing. Specifically, the number of grooves after desizing decreases significantly, the width of the grooves increases, and the depth of the grooves becomes slightly larger; however, the change is not obvious. The decrease in the number of surface grooves reduces the surface roughness of the CF, and the width and depth of the grooves both increase to an extent after the sizing agent layer covering the CF grooves is removed.

### 3.2. Physicochemical Characterization and Surface Energy of CFs

**Figure 4:**
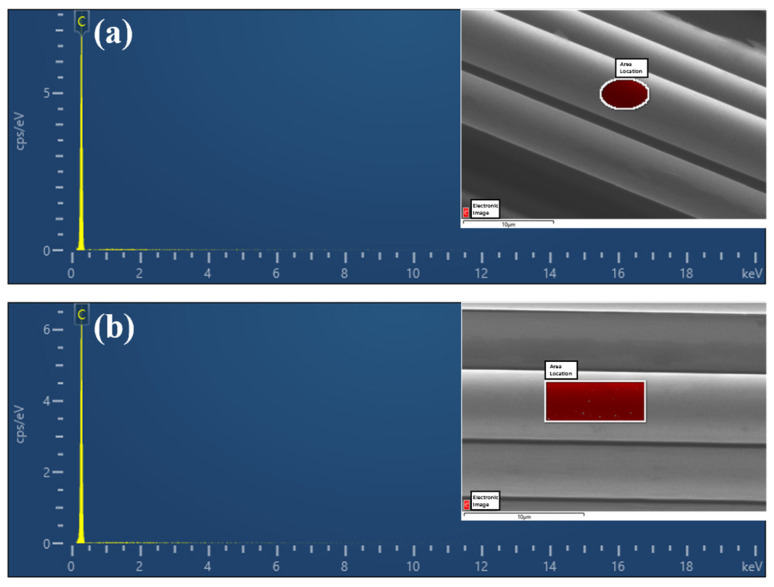
EDS test results for (**a**) CF800 and (**b**) CF800Q.

The element content of the CF surface was tested by EDS, with the results shown in Figure 4. It can be seen that the surfaces of both CF800 and CF800Q are dominated by elemental C, reflecting the chemical composition characteristics of CFs. The surface C element content (atomic percentage) of CF800 is 98.92% and that of CF800Q is 99.75%. These results are in line with expectations, as the surface of CF800 contains sizing agents which contain non-C components. The introduction of the sizing agent causes a decrease in the surface C content of CF800. After removal of the sizing agent (CF800Q), the corresponding non-C content decreases, resulting in the EDS test presenting a higher C content.

**Figure 5:**
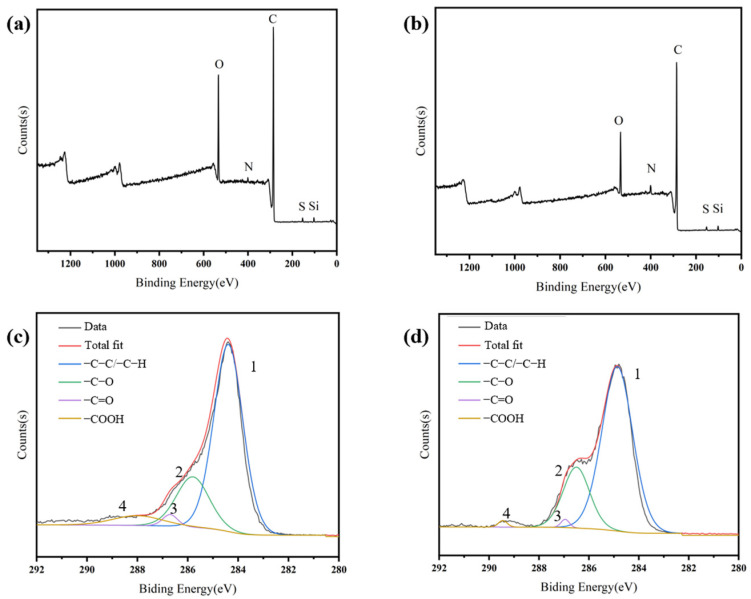
XPS test results of (**a**) CF800, (**b**) CF800Q, (**c**) C 1s of CF800, and (**d**) C 1s of CF800Q.

The active functional groups on the surface of the CFs and the composition of the sizing agent affect the interfacial strength of the resulting composites. In this section, the elemental and functional group compositions on the surface of the CFs were analyzed, and group compositions with different surface properties and proportions were investigated by full-spectrum scanning of the CF surface. From Figure 5a,b and Table 3 it can be seen that both CF800 and CF800Q contain several elements, including C, O, N, Si, and S. In comparison, the content of C on the surface of CF800Q increases significantly after desizing, while the content of other non-C elements decreases, leading to reduced surface chemical activity.

In order to more finely characterize the surface C states of CF800 and CF800Q, the C 1s peaks were subjected to detailed chemical analysis; the results are shown in Figure 5c,d and Table 4. It can be seen that the ratio of -C-C/-C-H on the surface of CF800Q obviously increased after desizing, while the ratio of other active C decreased, bringing about a decrease in surface chemical activity. Taken together, both EDS and XPS confirm that the presence of the sizing agent increases the CF surface chemical activity for CF800, which theoretically facilitates the interfacial bonding of CF/epoxy.

The contact angle test can reflect the surface energy of the CF and provide support for subsequent analysis of the interfacial bonding properties. Thus, the contact angle and surface energy ($\gamma_{s}$) of the CF samples, including the polar component ($\gamma_{s}^{p}$) and dispersive component ($\gamma_{s}^{d}$), were investigated. Here, the method from Young, Fowkes, Owens, and Wendt for measuring the surface energy of solids [44,45,46] was applied. The surface energy of the sample was calculated according to the following equation [47].

(4) $$\gamma_{l}(1+\cos\theta )=2(\sqrt{\gamma_{l}^{p}{\cdot \gamma }_{s}^{p}}+\sqrt{\gamma_{l}^{d}{\cdot \gamma }_{s}^{d}})$$

In the above equation, θ is the measured contact angle; $\gamma_{l}$, $\gamma_{l}^{p}$, and $\gamma_{l}^{d}$ need to be known for the test liquids used to contact the surface. In this work, deionized water, ethylene glycol, and formamide were chosen for testing. A linear equation with the slope and intercept is then applied to obtain the dispersive and polar components of the solid surface energy, and $\gamma_{s}$ can be calculated by adding $\gamma_{s}^{p}$ and $\gamma_{s}^{d}$. The results for the contact angle and $\gamma_{s}$ are illustrated in Table 5.

From the test results, it can be seen that the $\gamma_{s}$ of CF800 is higher than that of CF800Q, i.e., the presence of the sizing agent increases the $\gamma_{s}$ of the CFs. As a higher $\gamma_{s}$ is beneficial for CF/epoxy interfacial bonding, sizing is favorable to increase the interfacial binding property of CF/epoxy.

### 3.3. Microphase Structure of AC53X

**Figure 6:**
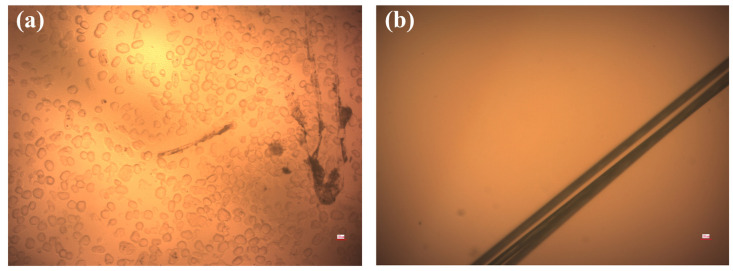
Microphase structures of (**a**) AC53X-1 and (**b**) AC53X-2.

It is very important to make structural observations of the resin, as the structure of the resin directly determines the fiber/resin interface properties. Therefore, the phase structure of the resin was observed. In order to investigate the effects of different phase structures on the interfacial bonding characteristics of the CF/epoxy, two kinds of AC53X-series epoxy resin, numbered AC53X-1 and AC53X-2, were used in this work. To characterize the microphase structure of the resin, a small amount of resin was uniformly coated on a glass slide and the microphase structure was observed by optical microscope. In Figure 6a, it can be seen that the second-phase resin is spherically distributed in the epoxy resin phase to form a monodisperse phase, which forms a two-phase coexistence structure. This resin was added at a level of 12.5%. In Figure 6b, only a single color of resin can be seen, and the resin has a single-phase structure.

### 3.4. Micro-Interfacial Performance of CF/Epoxy Composites

**Figure 7:**
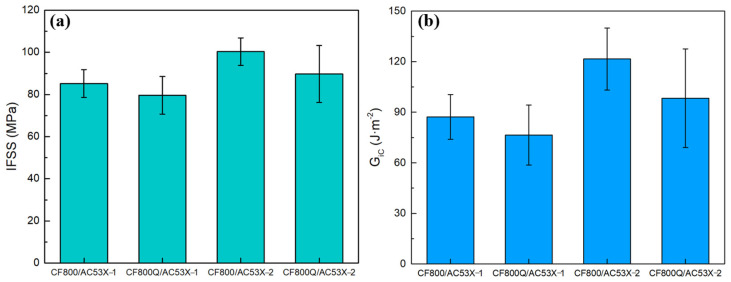
Interfacial properties of the CF and epoxy: (**a**) IFSS and (**b**) *G_ic_*.

The interfacial properties of the CF and epoxy were tested after the structures of the two systems were investigated separately, with the IFSS results shown in Figure 7. Figure 7a shows that the IFSS of CF800Q/AC53X-1 after desizing is lower than that of the corresponding sizing system. This result indicates that the presence of the sizing agent has a positive impact on the interfacial bonding between the CF and epoxy. This is due to the fact that the main component of the sizing agent is epoxy, which can form a good bond with the epoxy component in the matrix resin and form a strong interface after curing.

On the other hand, it is worth noting that the IFSS of CF800/AC53X-2 was higher than that of CF800/AC53X-1. The enhancement value was 17.7%. The *G_ic_* results reflect the same pattern as the IFSS results. Comparing the experimental results in Section 3.3, it can be seen that the introduction of the second phase into the resin causes a decrease in the interfacial bonding properties between the CF and epoxy. At the same time, a similar pattern is observed for the desized CFs. This phenomenon can be explained based on the fracture micromorphology of the specimens after the destruction of the interfacial bonding between the CF and resin.

**Figure 8:**
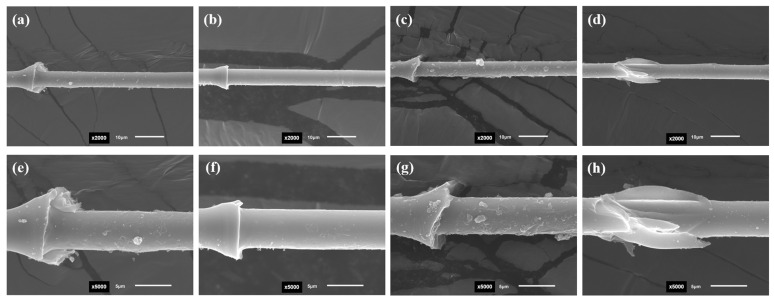
SEM images of (**a**,**e**) CF800/AC53X-1, (**b**,**f**) CF800Q/AC53X-1, (**c**,**g**) CF800/AC53X-2, and (**d**,**h**) CF800Q/AC53X-2 after the IFSS test.

The SEM results illustrate that the interfacial disruption of the specimens of different systems shows different micromorphological characteristics. Figure 8a,c shows CF800/AC53X-1 without desizing, while Figure 8c,g shows CF800/AC53X-2 without desizing. It can be seen that after the IFSS test the surface of the CF shows a “rough” physical characteristic, which reflects the fragmentation of the resin is during the loading process. As a result, some part of the resin is displaced by the interfacial disruption and some part remains on the surface of the CF. This phenomenon indicates better interfacial bonding between CF and epoxy. In addition, by comparing Figure 8e,g it can be clearly seen that the CF/epoxy bonding of CF800/AC53X-2 is better, which corresponds well with the IFSS data. Figure 8b,f shows CF800Q/AC53X-1 with the sizing agent removed, while Figure 8d,h shows CF800Q/AC53X-2 with the sizing agent removed. It can be seen that the surfaces of both CFs exhibit a “smooth” physical characteristic after the IFSS test, which reflects that the resin is basically displaced by the interfacial disruption during the loading process. There is almost no resin left on the surface of the CF. This phenomenon indicates that the interfacial bonding between the CF and epoxy is poor, corresponding to lower IFSS values.

### 3.5. IFSS after Hygrothermal Treatment

**Figure 9:**
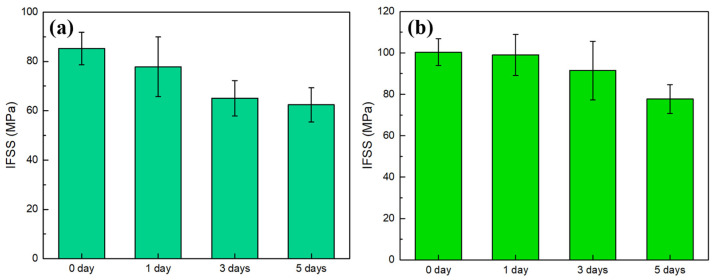
IFSS results after hygrothermal treatment of (**a**) CF800/AC53X-1 and (**b**) CF800/AC53X-2.

In order to study the effect of resins with different phase structures on the interfacial bonding strength of the CF/epoxy resin under a hygrothermal environment, two systems with relatively better performance (CF800/AC53X-1 and CF800/AC53X-2) were subjected to water-immersion aging treatment at 71 °C for one day, three days, and five days. The specimens were taken out at the corresponding times for IFSS tests, and the experimental results are shown in Figure 9.

The results illustrate that while the interfacial properties of the two systems after hygrothermal aging both show a decreasing trend with the growth of aging time, there are significant differences in their specific patterns of change. As shown in Table 6, for the CF800/AC53X-1 system, the decline of IFSS with the increase of aging time is first fast and then slow; after one day, the performance decreases by 8.7%, after three days, the performance decreases by 15.0%, and from the third day to the fifth day the performance decreases by 3.1%, basically maintaining a relatively stable level. On the other hand, for the CF800/AC53X-2 system, the decreasing trend of IFSS with the increase of aging time is slow and then fast; the performance decreases by only 1.4% after one day, then decreases by 7.4% after three days, and from the third day to the fifth day the performance decreases significantly by 13.7%. Comparing the change rule of their performance, CF800/AC53X-2 has significantly stronger resistance to moisture and heat aging than CF800/AC53X-1. After the hygrothermal treatment, both the IFSS values and performance retention rate of CF800/AC53X-2 show higher levels.

**Figure 10:**
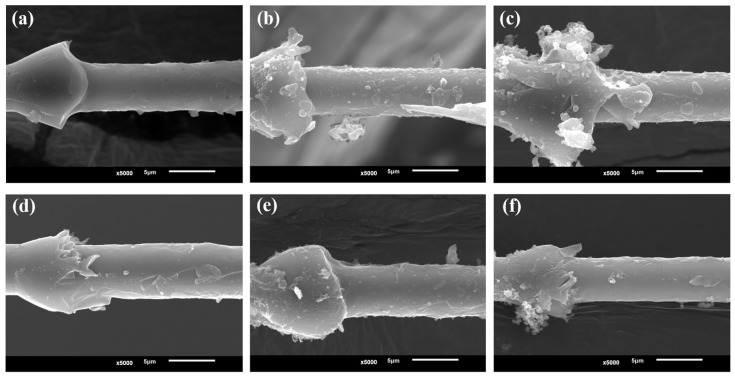
SEM images of samples after IFSS test: (**a**) CF800/AC53X-1, one day; (**b**) CF800/AC53X-1, three days; (**c**) CF800/AC53X-1, five days; (**d**) CF800/AC53X-2, one day; (**e**) CF800/AC53X-2, three days; (**f**) CF800/AC53X-2, five days.

In order to investigate the reason for the different patterns of change in the properties of the two systems, SEM analysis was carried out on the fracturing of the specimens after the IFSS test. The results are shown in Figure 10. It can be seen that there are obviously more defects in the resin of CF800/AC53X-1 due to the introduction of the second-phase resin, and that this phenomenon becomes more obvious with the increase in the number of days of hygrothermal treatment. On the other hand, this phenomenon does not occur in the single-phase CF800/AC53X-2 system. It can be inferred that the introduction of the second-phase resin brings about more hidden defect sources; under the hygrothermal environment, water molecules can diffuse inside the resin along the interfacial paths between the two resins, resulting in defects such as cracks and voids, and as a consequence the overall performance of the composite becomes degraded. In future work, further research on the compatibility of double-phase resins is needed in order to strengthen the interfacial bonding between the two kinds of resins. Therefore, the mechanical properties of the CF800/AC53X-1 and CF800/AC53X-2 systems will be researched in depth.

## 4. Conclusions

In this work, the CF and resin properties of two new composite systems, CF800/AC53X-1 and CF800/AC53X-2, were investigated, revealing the changes in the interfacial properties and environmental resistance of the resin matrix composites after the addition of the second phase. The following conclusions were obtained in the course of this study:

(1) The surface of CF800 shows a smooth characterization; after desizing, the surface roughness shows no difference, the number of grooves decreases, and the width and depth of the grooves slightly increase.
(2) The C content of CF800 rises after desizing, the ratio of inactive -C-C/-C-H rises, $\gamma_{s}$ decreases, and the sizing process has the positive effect of increasing the interfacial bonding properties of the CF/epoxy.
(3) The microphase structure of the resin samples shows that AC53X-1 has a double-phase structure and AC53X-2 has a single-phase structure.
(4) The IFSS of CF800Q/AC53X-1 is lower than that of the corresponding sizing system, indicating that the presence of the sizing agent is beneficial to the interfacial bonding between CF and epoxy. The IFSS of CF800Q/AC53X-2 is higher than that of CF800Q/AC53X-1, showing that the introduction of the second phase in the resin reduces the interfacial bonding performance between the CF and epoxy.
(5) After hygrothermal treatment, the IFSS value and property retention of CF800/AC53X-2 show a high level. The introduction of the second-phase resin brings about more hidden defect sources, allowing water molecules to diffuse along the interfacial paths between the two resins, which in turn creates defects and brings about a consequent reduction in the interfacial performance of the composite.

## Figures and Tables

**Table 1:** Roughness values of CF800 and CF800Q.

Sample	R_q_/nm
CF800	11.06 ± 7.11
CF800Q	11.19 ± 6.83

**Table 2:** Structural characteristics of the grooves.

Sample	Number of Grooves	Width of Grooves (nm)	Depth of Grooves (nm)
CF800	122	135	12
CF800Q	96	176	15

**Table 3:** Elemental compositions of the CF surfaces.

Name	Atomic %	
	CF800	CF800Q
C 1s	80.87	83.25
O 1s	16.48	11.72
N 1s	0.98	3.34
Si 2p	1.59	1.65
S 2p	0.07	0.04

**Table 4:** Functional group compositions of C 1s on the surface of CFs.

Name	Peak	B.E./eV	C 1 s Peaks in Different States B.E. (P.C., %)	
			CF800	CF800Q
-C-C/-C-H	1	284.39	70.25	74.48
-C-O	2	285.8	21.34	23.70
-C=O	3	286.7	2.20	1.00
-COOH	4	288	6.21	0.82

**Table 5:** Contact angle and surface energy of CF800 and CF800Q.

Sample	θ Water/°	θ Ethylene Glycol/°	θ Formamide/°	$\gamma_{s}$/mJ·m^−2^	$\gamma_{s}^{d}$/mJ·m^−2^	$\gamma_{s}^{p}$/mJ·m^−2^
CF800	75.86	61.95	57.45	29.95	10.17	19.79
CF800Q	81.76	62.31	68.96	26.02	14.66	11.36

**Table 6:** IFSS performance retention after hygrothermal treatment.

Performance Retention/%	1 Day	3 Days	5 Days
CF800/AC53X-1	91.3	76.3	73.2
CF800/AC53X-2	98.6	91.2	77.5

## Data Availability

The data presented in this study are available upon request from the corresponding author. The data are not publicly available due to the fact that they are also part of an ongoing study.

## Supplementary Materials

The following supporting information can be downloaded at: https://www.mdpi.com/article/10.3390/ma17061323/s1, Figure S1: Structure of epoxy resin of AC53X; Figure S2: Structure of curing agent of AC53X; Table S1: Properties of CF800; Table S2: Properties of AC53X.
