# Supplementary material for: Influence of the Second-Phase Resin Structure on the Interfacial Shear Strength of Carbon Fiber/Epoxy Resin
Source: Materials (Basel). 2024 Mar 13;17(6):1323. doi: 10.3390/ma17061323 (PMC10972230; doi:10.3390/ma17061323)
Supplement: Supplementary file 1 [file materials-17-01323-s001.zip › materials-2906269-supplementary.pdf]

Influence of the Second-Phase Resin Structure on the Interfacial Shear Strength of Carbon Fiber/Epoxy Resin

Supplementary Materials

Hansong Liu [1], Jinsong Sun [1], Lianwang Zhang [1], Zhaobo Liu [2], Chengyu Huang [2], Mingchen Sun [2], Ziqi Duan [3], Wenge Wang [3], Xiangyu Zhong [1,*] and Jianwen Bao [1,3,*]

1 AVIC Manufacturing Technology Institute Composite Technology Center, Beijing, China; liuhansongzhfc@foxmail.com (H.L.); sunjsbuaa@163.com (J.S.); zhanglian51@163.com (L.Z.)

2 School of Materials Science and Engineering, Beihang University, Beijing, China; lzbbuaa@163.com (Z.L.); huangchengyu@buaa.edu.cn (C.H.); smc703@126.com (M.S.)

3 AVIC Composite Corporation Ltd., Beijing, China; dzq970920@163.com (Z.D.); 15966308692@163.com (W.W.)

* Correspondence: xyzhong2003@sohu.com (X.Z.); baojianwen@sohu.com (J.B.)

Table S1. Properties of CF800.

Fiber Type	Filament Count	Tensile Strength/MPa	Tensile Modulus/GPa	Strain at Failure/%	Weight/Length /g·km^{-1}
CF800	12K	5641	295	1.93	447

Table S2. Properties of AC53X.

Resin Type	Structure-Epoxy resin	Epoxy value /mol·100g^{-1}	Structure-Curing agent	Amine value /mgKOH·g^{-1}	Resin/ curing agent mass ratio	Content of RS2000/%
AC53X-1	Figure S1	0.80~0.87	Figure S2	62.1	100:52	12.5
AC53X-2	Figure S1	0.80~0.87	Figure S2	62.1	100:52	0

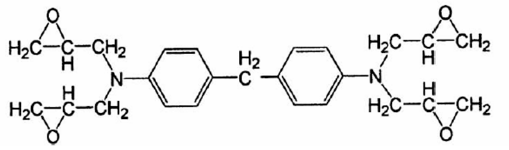

Figure S1. Structure of epoxy resin of AC53X.

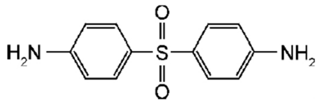

Figure S2. Structure of curing agent of AC53X.
